# Biological activities of *Viscum tuberculatum* aqueous leaf extract

**DOI:** 10.1080/13880209.2022.2151021

**Published:** 2022-12-20

**Authors:** Abraham Yirgu, Yalemtsehay Mekonnen, Amelework Eyado, Alessia Staropoli, Francesco Vinale

**Affiliations:** a Ethiopian Forest Development, Central Ethiopia Center, Addis Ababa, Ethiopia; b Department of Biology, College of Natural and Computational Sciences, Addis Ababa University, Addis Ababa, Ethiopia; c Department of Agricultural Sciences, University of Naples Federico II, Portici, Italy; d Institute for Sustainable Plant Protection, National Research Council, Portici, Italy; e Department of Veterinary Medicine and Animal Production, University of Naples Federico II, Naples, Italy

**Keywords:** Antibacterial activity, anti-inflammatory activity, acute toxicity, metabolite, *Staphylococcus aureus*, *Escherichia coli*, *Pseudomonas aeruginosa*, Ethiopia

## Abstract

**Content:**

Plant-based natural products have served as sources of remedies against pathogenic microorganisms. Although the biological activities of *Viscum* (Santalaceae) species are widely recognized, there is no scientific evidence for *Viscum tuberculatum* A. Rich. in Ethiopia.

**Objective:**

To investigate the antimicrobial, acute toxicity, anti-inflammatory properties and phytochemical constituents of an aqueous extract of *V. tuberculatum* from Ethiopia.

**Materials and methods:**

The antibacterial activity of the aqueous leaf extract of *V. tuberculatum* was tested against *Escherichia coli*, *Pseudomonas aeruginosa* and *Staphylococcus aureus.* The minimum inhibitory concentration (MIC) and minimum bactericidal concentration (MBC) of this extract were determined using the broth macrodilution method. The acute toxicity and anti-inflammatory effects of the extract were investigated using standard procedures on female and male white albino mice, aged 8 and 10 weeks, respectively. The phytochemical constituents of *V. tuberculatum* were determined using LC–MS QTOF.

**Results:**

The MIC and MBC values against *S. aureus* were found to be 6.25 and 100 mg/mL. The LD_50_ value was more than 2000 mg/kg body weight of the mouse. The 400 mg/kg dose exerts 87% inhibition after 5 h of carrageenan injection. Twenty-five different metabolites, mainly flavonoids, phenolic acids and alkaloids, were identified.

**Conclusions:**

These findings demonstrate the potential antimicrobial and anti-inflammatory potential of the aqueous extract of *V. tuberculatum*.

## Introduction

Antibiotic-resistance has caused a considerable threat in the treatment of human and animal infectious diseases (Leonard et al. 2022). The problem with the multi-drug resistance profiles of the most commonly prescribed drugs is well recognized in several African countries, including Ethiopia (Efa et al. 2019). A recent literature review on the status of antimicrobial resistance in Ethiopia revealed a high prevalence of resistance to common and clinically important bacteria, including *Staphylococcus aureus* (Fujita et al. 2022). The emergence of antimicrobial resistance even to third generation drugs has sparked a great interest in discovering new antimicrobial agents from natural sources, such as plants (Malada et al. 2022).

*Viscum* L. genera (Santalaceae, Viscaceae), commonly known as mistletoe, comprises about 100 species of mistletoe. These species are distributed in Africa, Asia, Australia and Europe (Polhill and Wiens 1998). Several *Viscum* species are rich in diverse biologically active secondary metabolites that treat various health ailments, including antimicrobial activities against various pathogenic bacteria (Amabeoku et al. 1998; Erturk et al. 2003; Ahmmed et al. 2013; Assaf et al. 2013; Nag et al. 2016).

*Viscum tuberculatum* A. Rich. (syn. *V. camporum*, *V. holstii*, *V. spragueanum* and *V. stuhlmannii*) is a hemi-parasitic shrub that grows on a wide range of host plants (Gilbert 1989; Polhill and Wiens 1998; Yirgu and Tesfaye 2018). It is distributed in several African countries (Polhill and Wiens 1998). In traditional medicine, it treats pneumonia and the liver (Idris 2004), diarrhoea, vomiting, fevers, and headaches (Issa 2015), cough (Demie et al. 2018), malaria, and acute diseases (Abdeta et al. 2020). It also serves as food for wild animals (Bussmann 2006). The phytochemical constituents and biological activity of *V. tuberculatum* are not well known. Therefore, this study investigated the phytochemical constituents, antimicrobial activity, acute toxicity and anti-inflammatory activities of the aqueous extract of *V. tuberculatum* from Ethiopia.

## Materials and methods

### Plant collection and identification

Matured, fresh and healthy leaves of *V. tuberculatum* were collected from *Teclea nobilis* Delile in the Shashemene District of Central Ethiopia between April and June 2018. The taxonomic identity of *V. tuberculatum* was authenticated by Mr. Melaku Wondafrash a herbarium specialist at Addis Ababa University National Herbarium (ETH), Ethiopia. A voucher specimen (AY24) was deposited at ETH.

### Preparation of extracts

The leaf samples of the mistletoe were washed in running tap water, air-dried under shade at room temperature, and finely powdered using an electric grinder. The dried powder (20 g) was macerated in 200 mL of sterile distilled water at room temperature. The solution was agitated on a daily basis for three consecutive days, filtered through Whatman no. 1 filter paper and dried with a lyophilizer (CHRIST^®^, Osterode, Germany).

### Antibacterial assay

#### Test microorganisms

The bacteria (*Escherichia coli* ATTC 25922, *Pseudomonas aeruginosa* ATTC 27853 and *Staphylococcus aureus* ATCC 25923) were obtained from the Ethiopian Public Health Institute (EPHI), Addis Ababa, Ethiopia. These bacteria were sub-cultured overnight in nutrient agar medium at 35 ± 2 °C. Four to five isolated colonies of the same morphological type of bacteria were picked with a sterile loop and dissolved in a 0.9% physiological saline solution.

#### Antibacterial activity of plant extracts

The antimicrobial activity of *V. tuberculatum* was tested using Whatman filter paper no. 1 disks impregnated with plant extract as described by Hudzicki (2009) and Ghaedi et al. (2015). The turbidity of the bacterial suspension was adjusted to 0.5 McFarland standards (1–2 × 10^8^ CFU/mL). Bacterial suspension (100 µL) was transferred and gently spread over the Mueller–Hinton Agar (MHA) surface using a sterile cotton swab. Disks were aseptically placed on an MHA plate inoculated with each of the test bacteria, and incubated at 35 ± 2 °C for 16–18 h.

#### Determination of the minimum inhibitory concentration (MIC)

The MIC of *V. tuberculatum* against bacterial strains was determined by a rapid *p*-iodonitrotetrazolium chloride (INT; ROTH, Carl Roth GmbH Co., Karlsruhe, Germany) colorimetric assay using the macrodilution method (Chukwujekwu and van Staden 2016; Mfengwana and Mashele 2016). A two-fold serial dilution of plant extract (1 mL) ranging from 100 up to 0.098 mg/mL was aseptically transferred into 10 sterile test tubes containing 2 mL of Mueller–Hinton Broth (MHB). Test tubes no. 11 (containing MHB and test bacterium) and 12 (containing MHB, test bacterium and standard reference drug) were prepared as negative and positive controls, respectively. Finally, a 100 µL bacterial suspension adjusted to 0.5 McFarland standards was added to each of these 12 test tubes. These test tubes were incubated at 35 ± 2 °C for 18 h (Smith et al. 2020). Then, 60 µL of INT (2 mg/mL of INT in sterile distilled water) was added to all test tubes and further incubated for 2 h at 35 ± 2 °C. Active bacterial growth reduces the yellow INT dye to a pink colour (Sati et al. 2019). The MIC was determined as the lowest concentration of the aqueous extract that resulted in the absence of bacterial growth in the test tube detected by the unaided eye and the absence of colour change (Eloff 1998).

#### Determination of minimum bactericidal concentration (MBC)

The MBC was determined using the macrodilution broth method as described by Yilmaz (2012) with slight modification. Aliquots (100 µL) of each gradient concentrated solution from the MIC activity were transferred into 12 test tubes filled with fresh MHB medium. These tubes were incubated at 35 ± 2 °C for 48 h. The MBC endpoint was considered as the lowest concentration of plant extract that showed no colour change after the addition of INT, which showed a complete absence of microbial growth.

### Ethical issues

The use of Swiss albino mice in the investigation of acute toxicity and anti-inflammatory activity of *V. tuberculatum* was approved by the College of Natural and Computational Sciences Institutional Review Board (CNS-IRB) of Addis Ababa University (Ref: CNCSDO/424/13/2021), Ethiopia. All experimental procedures conducted in the study were compliant with the internationally accepted principles for laboratory animal use and care.

### Acute oral toxicity evaluation

The acute toxicity of the aqueous extract of *V. tuberculatum* was determined according to the up-and-down procedure of Organization for Economic Cooperation and Development (OECD) Guidelines No. 425 (OECD 2008) with slight modification. Twenty-five Swiss female albino mice (healthy, nulliparous, non-pregnant and 8–10 weeks old) were obtained from EPHI Animal House. These mice were randomly divided into five groups (*n* = 5) based on body weight (bw). They were acclimatized for five days to a 12 h light/dark cycle and had free access to food and water. All the mice fasted for food with free access to water the night before the experiment. Mice in groups I and II served as controls and received tap water and physiological saline (vehicle) solution, respectively. Groups III, IV and V mice received 175, 550 and 2000 mg/kg bw of dry plant extract dissolved in physiological saline, respectively. Food was provided after 2 h of dosing. The signs of toxicity, behavioural changes and mortality of mice were observed during the first 30 min, 4 h, 24 h, and once a day for 14 successive days after administration of the treatments.

The bw of mice was taken before treatment (day 0), on day 7, and on day 14 after the administration of crude plant extract. At the end of 14 days of observation, the liver, kidneys, heart and lungs of each mouse were removed and weighed. The 50% lethal dose (LD_50_) was estimated. The percent body and the relative vital organ weights of animals were calculated as stated by Porwal et al. (2017).

$$\text{\% weight gain}=[\frac{\left(\text{final bw}-\text{initial bw}\right)}{\text{final bw}}]\times 100$$

$$\text{Relative organ weight}=[\frac{\text{organ weight}}{\text{bw of animals on sacrifice day}}]\;\times \;100$$

### Anti-inflammatory activity

The anti-inflammatory activity of the plant extract was evaluated on carrageenan-induced inflammatory paw oedema, as described by Adnan et al. (2019) and Bezerra et al. (2017) with some modifications. Swiss male albino mice were divided based on bw into four groups of six animals in each. These animals (healthy, 8–10 weeks old) were deprived of food overnight and allowed free access to water prior to the experiment. Group I mice were treated with vehicle as a negative control, group II with Indomethacin (10 mg/kg bw) served as a positive control, and groups III and IV received 200 and 400 mg/kg bw of plant extract, respectively. After 1 h of oral administration of these substances, oedema was induced by injecting a suspension of 0.1 mL of 1% carrageenan (w/v) in physiological saline into the sub-plantar tissue of the right hind paw of each mouse. The initial thickness of the hind paw was measured before the injection of carrageenan suspension. Subsequent measurement of paw thickness was measured at 1 h intervals for 5 h using a Digital LCD Vernier Caliper Gauge micrometer. The percent inhibition of paw volume was calculated using the following equation (Boutennoun et al. 2017).

% inhibition=[((Vt−Vo) control−(Vt−Vo) treated group)/(Vc−Vo) control]×100

where Vt and Vo denote the right hind paw thickness volume (in milliliters) at time *t* and before carrageenan injection, respectively.

### Liquid chromatography–mass spectrometry (LC–MS) analysis

The crude aqueous leaf extract of *V. tuberculatum* with active antimicrobial activity against a test pathogen was analysed on an Agilent HP 1260 Infinity Series liquid chromatograph (Agilent Technologies, Santa Clara, CA) equipped with a diode array detector (DAD) system (Agilent Technologies, Santa Clara, CA) and coupled to a quadrupole-time of flight (Q-TOF) mass spectrometer model G6540B (Agilent Technologies, Santa Clara, CA) with a dual electrospray ionization (ESI) source (Agilent Technologies, Santa Clara, CA). Chromatographic separation was conducted using a Zorbax Eclipse Plus C-18 column (4.6 × 50 mm, with 3.5 µm particle size), SepaChrom Srl, Rho, Italy) at 37 °C. The elution solvent consists of eluent A: 0.1% (*v/v*) formic acid (FA) in water and, eluent B: 0.1% (v/v) FA in acetonitrile at a flow rate of 0.5 mL/min. The gradient elution was started with 5% B to 70% B for the first 4 min, 70–80% B in 3 min, 80% B to 100% B in 2 min, 100% B in 4–5 min, and equilibrating at 95% A, 10–14 min. UV spectra were collected by DAD every 0.4 s from 190 to 750 nm with a resolution of 2 nm. MS parameters were set using Mass Hunter Qualitative Analysis Software version B.06.00 (Agilent Technologies, Santa Clara, CA), raw data were processed based on retention time and characteristic behaviour of MS, including the exact mass, quasimolecular ions and in-source fragmentation, data were compared with known compounds in an in-house plant database and existing literature. The acquisition was performed from 100 to 1700 *m/z*, with three scans per second. Positive identifications of plant metabolites were considered for analysis if the compound was detected with a mass error of below 10 ppm and with a sufficient score. Analytical and LC–MS grade chemicals were used throughout the metabolic profiling.

### Statistical analysis

Experimental results on the antimicrobial activity, anti-inflammatory effects and acute toxicity tests are presented as mean ± standard deviation (SD). These data were subjected to analysis of variance (ANOVA) using SAS v. 9 Statistical Software (Cary, NC). A Tukey’s HSD *post hoc* test for multiple comparisons was performed to detect the differences between treatment means. A *p*≤ 0.05 was considered statistically significant. Descriptive statistical analysis was used for non-parametric data.

## Results

### Anti-bacterial activity

**Figure 1.**
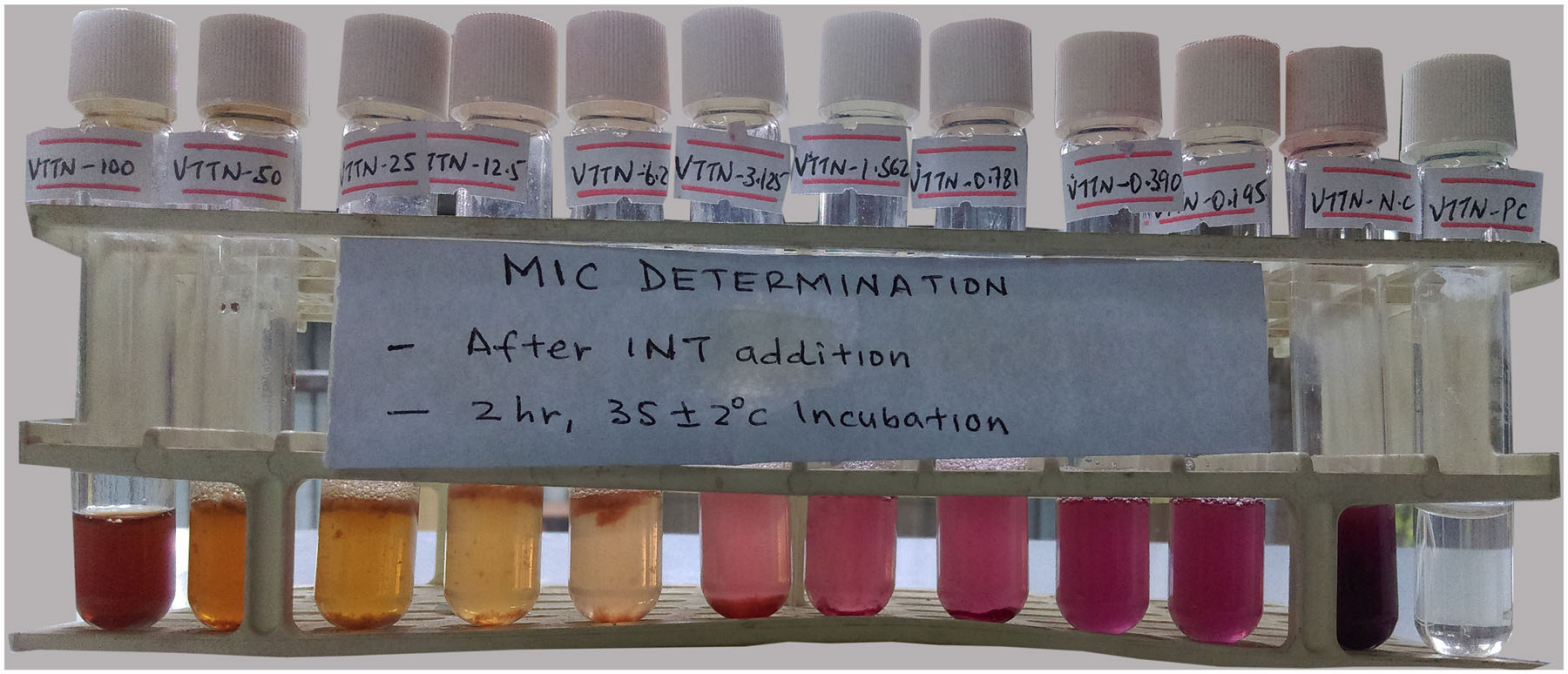
MIC determination of crude aqueous extract of VTTN against *S. aureus.*

**Figure 2.**
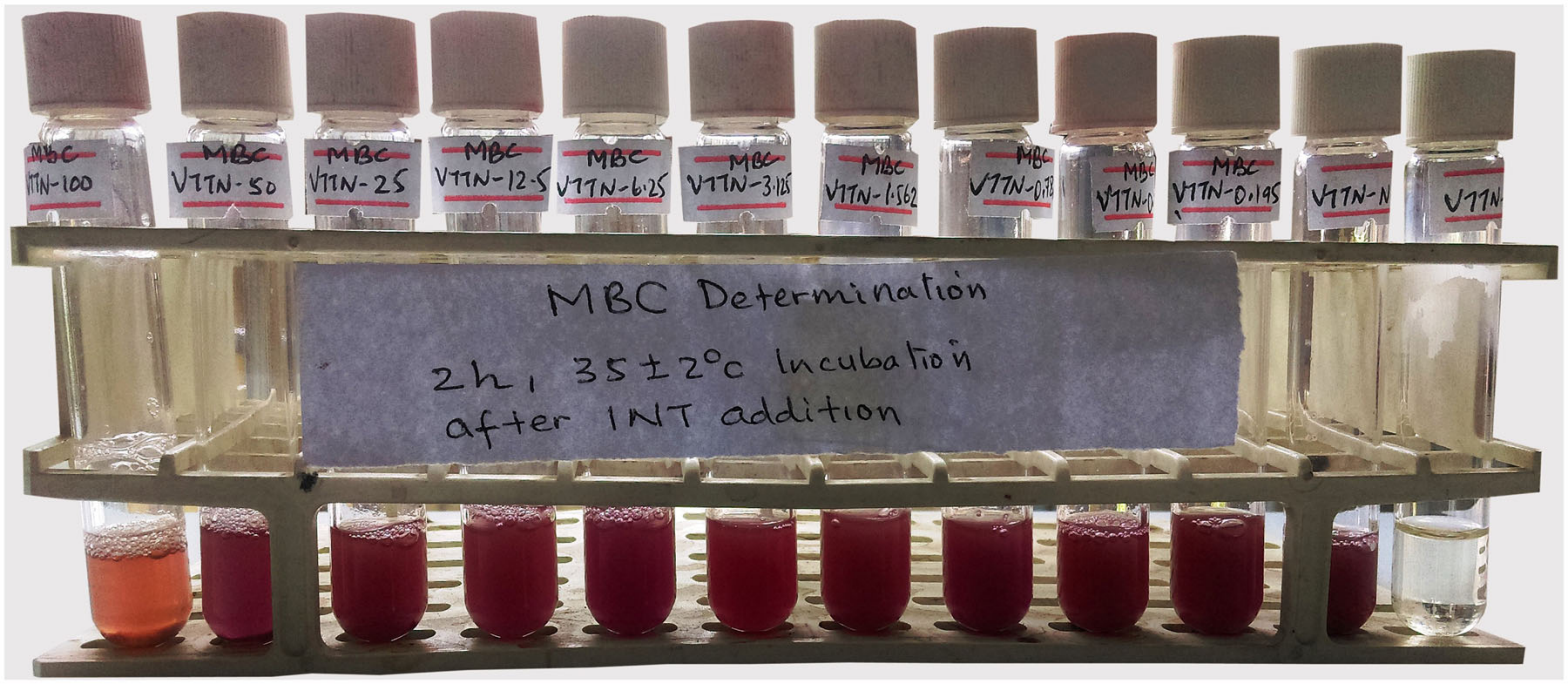
MBC determination of crude aqueous extract of VTTN against *S. aureus.*

The aqueous leaf extract of *V. tuberculatum* inhibited the bacterial growth of *E. coli*, *P. aeruginosa* and *S. aureus.* The macrodilution determination of the MIC and MBC was conducted against *S. aureus* for its higher susceptibility compared with *E. coli* and *P. aeruginosa*. Hence, erythromycin was selected as a standard antibacterial agent in determining the MIC and MBC of the plant extract against *S. aureus*. The MIC (Figure 1) and MBC (Figure 2) showed the growth inhibition of *S. aureus* at ≤6.25 and ≥100 mg/mL, respectively. The negative control in both MIC and MBC showed active growth, indicated by a colour change from yellow to pink. The standard drug (in test tube 12) inhibited the growth of *S. aureus* so that the solution was colourless. In contrast to the lower MIC value (≤6.25), the MBC test revealed *S. aureus* growth at all test concentrations, which indicated the MBC value is higher than 100 mg/mL.

### Acute oral toxicity

#### Behavioural changes, mortality and body weight gain of mice

The oral administration of three doses (175, 550 and 2000 mg/kg bw) of the aqueous *V. tuberculatum* extract did not show any behavioural changes compared to the controls. In addition, no cases of coma or mortality of mice were observed in the two-weeks period of observation. On the other hand, all mice showed a progressive weight gain during the two weeks of observation. Nevertheless, the weight gain in treated mice showed a non-significant difference compared with the control animals (Table 1).

**Table 1.** The body weight of mice before and after treatments with crude extract, vehicle and tap water.

Treatment	Mortality (dead/treated mice)	Body weight (g) (mean ± SD), *n* = 6		
		Baseline weight	7th day	14th day
Water	0/5	21.20 ± 2.59	24.56 ± 2.36	27.08 ± 2.03
Saline	0/5	21.40 ± 1.67	24.12 ± 2.67	25.34 ± 2.43
175 mg/kg	0/5	21.80 ± 1.92	24.50 ± 1.61	26.22 ± 1.68
550 mg/kg	0/5	22.40 ± 2.70	26.06 ± 3.29	27.92 ± 3.04
2000 mg/kg	0/5	23.80 ± 1.48	25.90 ± 1.22	29.12 ± 1.83

#### Effect of acute oral toxicity test on vital organ weight of mice

The effect of aqueous extract on the relative organ weights of the heart, lungs, liver and kidneys of all treated mice was determined at the end of the acute toxicity test. There was no significant difference (*p* > 0.05) in the organ weights of all the treated animals (Table 2). The visual inspection of these organs also revealed no evidence of injury to these vital organs at all doses.

**Table 2.** The relative vital organ to body weight ratio of mice after acute oral toxicity test.

Treatment	Heart	Lung	Liver	Kidney
Water	0.56 ± 0.06	0.83 ± 0.17	5.59 ± 0.40	1.17 ± 0.09
Saline	0.58 ± 0.08	0.78 ± 0.12	5.44 ± 0.62	1.24 ± 0.10
175 mg/kg	0.61 ± 0.06	0.82 ± 0.07	5.49 ± 0.43	1.08 ± 0.13
550 mg/kg	0.53 ± 0.05	0.74 ± 0.05	5.48 ± 0.48	1.22 ± 0.09
2000 mg/kg	0.62 ± 0.10	0.73 ± 0.07	5.98 ± 0.83	1.24 ± 0.10

### Anti-inflammatory effect

The *in vivo* anti-inflammatory activity of *V. tuberculatum* at 200 and 400 mg/kg bw in carrageenan-induced paw oedema is shown in Table 3. The extract caused dose-dependent inhibition of paw oedema in the 5 h of observation. The highest percent (i.e., 87%) inhibition of oedema was observed in the 400 mg/kg bw after 5 h after carrageenan injection. A significant difference in the percentage of inhibition between indomethacin and the plant extract occurred in the 200 mg/kg dose after 2 h of carrageenan injection. A similar significant difference occurred between the 200 mg/kg dose and the control after 4–5 h of carrageenan administration. The percentage inhibition of paw oedema in mice treated with indomethacin increased to 85% 5 h after being treated with carrageenan solution (Table 3).

**Table 3.** Thickness of paw oedema and inhibition percentage of paw oedema induced by carrageenan suspension.

Treatment	Dose	1 h	2 h	3 h	4 h	5 h
Control	–	1.21 ± 0.20 (0.08)	1.34 ± 0.27 (0.08)*	1.30 ± 0.14 (0.08)*	1.37 ± 0.09 (0.08)*	1.27 ± 0.06 (0.08)*
Indomethacin	10	0.98 ± 0.33 (0.08)	0.67 ± 0.21 (0.08) (50)	0.55 ± 0.23 (0.08) (57.69)	0.37 ± 0.20 (0.08) (72.99)	0.19 ± 0.17 (0.08) (85.04)
VTTN	200	1.18 ± 0.33 (0.11)	1.25 ± 0.25 (0.11)*	0.99 ± 0.51 (0.11) (23.85)	0.61 ± 0.34 (0.11)^§^ (53.28)	0.39 ± 0.21 (0.11)^§^ (64.57)
	400	0.68 ± 0.38 (0.11)	0.71 ± 0.37 (0.11)^§^ (47.01)	0.49 ± 0.18 (0.11)^§^ (62.31)	0.30 ± 0.17 (0.11)^§^ (74.45)	0.13 ± 0.11 (0.11)^§^ (84.25)

Mean ± SD (SEM) and percentage of inhibition.

* The mean difference is significant at the 0.05 level between indomethacin and the control and plant extract doses at the respective time of observation.

^§^ The mean difference is significant at the 0.05 level between the control and plant extract doses at the respective time of observation.

### Identification of metabolites by LC–MS

Twenty-five different metabolomes were identified in the aqueous leaf extract of *V. tuberculatum* (Table 4). Eighteen metabolomes were identified in the positive ionization mode and 14 in the negative ionization mode. Six compounds were commonly found in both modes of analysis. These 25 compounds were distributed into nine different classes of compounds, viz., flavonoids (13), phenolic acids (3), alkaloids (2), sugars (2), indoles (1), coumarins (1), lignans (1), carboxylic acids (1) and polyphenols (1). The retention times, mass data and molecular formulas of these metabolomes are listed in Table 4.

**Table 4.** LC–MS QTOF identified metabolites in the aqueous extract of *V. tuberculatum.*

Ionization mode	Compound	Formula	Monoisotopic mass (Da)	RT (min)	*m/z*	Peak area %
*Flavonoid derivatives*						
–	(–)-Epicatechin	C_15_H_14_O_6_	290.0792	2.14	289.0724	29.02
–	(–)-Epicatechin 3-*O*-gallate	C_22_H_18_O_10_	442.0903	5.25	441.0846	90.15
–	Catechin-(4α->8)-epicatechin-3-*O*-gallate	C_37_H_30_O_16_	730.1534	4.85	729.1462	95.85^a^
–	Quercetin 7-(6″-galloylglucoside)	C_28_H_24_O_16_	616.1067	5.07	615.1002	7.84
+	Robinetidinol (4α-6) catechin	C_30_H_26_O_12_	578.1422	1.94	579.1502	86.85^a^
–	Catechin 5,7,-di-*O*-gallate	C_29_H_22_O_14_	594.1017	5.42	593.0944	49.48^a^
+	Guibourtinidol-4α-ol	C_15_H_14_O_5_	274.0836	5.78	275.0909	66.63
–	Isokaempferide 7-glucoside	C_22_H_22_O_11_	462.1171	5.86	461.11	18.48
+	Isorhamnetin	C_16_H_12_O_7_	316.0583	5.51	317.0657	70.13
–	Isorhamnetin 3-galactoside	C_22_H_22_O_12_	478.1113	5.58	477.104	49.48^a^
–	Isovitexin 2″-*O*-(6‴-(*E*)-*p*-coumaroyl) glucoside	C_36_H_36_O_17_	740.1951	1.79	739.1879	13.11^a^
–	Myricetin 3-(2″-galloylrhamnoside)	C_29_H_26_O_16_	630.1237	5.42	629.1157	49.48^a^
+	Robinetinidol-(4α->8)-[7,8-dihydro-8-(2,4-dihydroxyphenyl)-7-hydroxy-6-(3,4,5-trihydroxyphenyl)-6H-pyrano[2,3-*f*]catechin]	C_45_H_38_O_18_	866.2051	1.96	867.2125	86.85^a^
*Phenolic acids derivatives*						
–	Quinic acid	C_7_H_12_O_6_	192.064	1.28	191.0567	39.41^a^
+	4-hydroxybenzoic acid	C_7_H_6_O_3_	138.0313	4.52	139.0388	71.07
–	Pyrocatechin	C_6_H_6_O_2_	110.0369	1.28	109.0296	39.41^a^
*Alkaloids derivatives*						
+	Trigonelline	C_7_H_7_NO_2_	137.0476	1.21	138.0548	21.07^a^
+	Calystegin A3	C_7_H_13_NO_3_	159.0896	1.56	160.0969	35.4
*Sugars*						
–	Maltose	C_12_H_22_O_11_	342.1167	1.24	341.1094	39.41^a^
+	Vicianose	C_11_ H_20_ O_10_	312.1063	1.22	313.0693	21.07^a^
*Indoles*						
+	Indole-3-acrylic acid	C_11_H_9_NO_2_	187.0629	1.84	188.0701	86.85^a^
*Coumarins*						
–	(+)-Marmesin	C_14_H_14_O_4_	246.0894	4.53	245.0821	95.85^a^
*Lignans*						
+	Ligballinol	C_18_H_18_O_4_	298.1212	6.23	299.1285	9.26
*Carboxylic acids*						
+	l-Pipecolic acid	C_6_H_11_NO_2_	129.0788	1.3	130.0861	21.07^a^
*Polyphenols*						
–	Gallic acid	C_7_H_6_O_5_	170.0213	1.81	169.0147	13.11^a^

^a^ Co-eluted compounds whose signals share the same peak area.

## Discussion

In this study, the aqueous leaf extract of *V. tuberculatum* inhibited the growth of *E. coli* ATTC 25922, *P. aeruginosa* ATTC 27853 and *S. aureus* ATCC 25923. A relatively higher inhibitory activity of the plant extract was observed against *S. aureus* (Gram-positive bacteria) than the two Gram-negative bacteria, which may be attributed to the presence of lipopolysaccharide in Gram-positive bacteria (Breijyeh et al. 2020). Previous studies have also showed the antimicrobial properties of *Viscum* species. For instance, the hexane, ethyl acetate, acetone and methanol extracts of *V. rotundifolium* L.f. showed antimicrobial activity against *E. coli*, *P. aeruginosa*, *S. aureus* and *Enterococcus faecalis* (Malada et al. 2022). The methanol extract of *V. articulatum* Burm. F. has also inhibited *S. aureus* and *E. coli* (Nag et al. 2016). In addition, the ethyl acetate, chloroform, ethanol, methanol and water leaf extracts of *V. album* L. inhibited *E. coli*, *P. aeruginosa*, *S. aureus* and other Gram-positive and Gram-negative bacteria (Hussain et al. 2011).

The acute toxicity study carried out using the 175, 550 and 2000 mg/kg bw doses did not reveal any observable behavioural changes or deaths of mice. The insignificant weight gain following the administration of the plant extract suggests the physiological wellness of mice (Belghoul et al. 2020). This indicates that the medium lethal dose (LD_50_) value of the orally administered *V. tuberculatum* is greater than 2000 mg/kg bw, which can classify this plant extract under category 5 of the Globally Harmonized System of Classification and Labeling of Chemicals (UN 2011). A previous study into the acute toxicity of methanolic leaf extracts of *V. capitellatum* Sm. revealed the safety of this extract up to 2000 mg/kg bw but toxic at 5000 mg/kg (Jadhav et al. 2022). In addition, the ethyl acetate–water extracts of *V. album* exhibited no mortality in animals at a dose of 5000 mg/kg (Pozdnyakov et al. 2021).

In the *in vivo* anti-inflammatory activity, a significantly higher anti-inflammatory activity of *V. tuberculatum* was shown at a 400 mg/kg dose induced by the carrageenan suspension. The studies on *V. album* (Murthuza and Manjunatha 2018), *V. articulatum* (Leu et al. 2006), *V. coloratum* (Hwang et al. 2006) and *V. cruciatum* Sieber ex Boiss. (Assaf et al. 2013) have also shown the anti-inflammatory properties of these *Viscum* species.

The phytochemical analysis of *V. tuberculatum* revealed 25 metabolites, belonging to different classes of natural compounds, such as flavonoid derivatives, phenolic acids, alkaloids, sugars, indoles, coumarins, lignans, carboxylic acids and polyphenols. Some of these natural products are recognized for their antimicrobial and anti-inflammatory activities. For instance, quinic acid (Adamczak et al. 2019) and gallic acid (Olmedo-Juarez et al. 2019) are known for their antimicrobial against *E. coli*, *P. aeruginosa* and *S. aureus* and anti-inflammatory activities in previous studies. In this study, they are found to account for 39.41 and 13.11% of the total abundance. 4-Hydroxybenzoic acid, which accounted for 71.07% of the total abundance in this study, has been reported to antimicrobial and fungicidal activity (Anand et al. 2019). Similarly, metabolites such as (–)-epicatechin (Bettaieb et al. 2016; Araujo et al. 2019), gallic acid (Li et al. 2017) and isorhamnetin (Tian et al. 2021) have exhibited both antimicrobial and anti-inflammatory properties. Epicatechin and isorhamnetin, which were identified in this study accounted for 29.02% and 70.13% of total abundance, respectively. The antioxidant activity of quinic acid (Karaman et al. 2021), catechin and gallic acid (Đorđević et al. 2018) were also reported in previous studies. Additionally, marmesin (which account for 95.85% of total abundance) has been reported for its potent anti-proliferative property (Dong et al. 2018), and trigonelline (that account for 21.07% of total abundance) has antidiabetic, antioxidant, anti-inflammatory and neuroprotective effects (Khalili et al. 2018), respectively.

## Conclusions and recommendations

*V. tuberculatum* is one of the most widely distributed mistletoes used in the treatment of various health ailments. Understanding the limitation of data on the haematology and pathology of experimental mice, this study provides the first scientific report on the antimicrobial and anti-inflammatory activities of the aqueous *V. tuberculatum* collected from *Teclea nobilis* in Ethiopia. The identified secondary metabolites are, in part, responsible for the biological activities of the plant. This highlights the importance of using the aqueous extract of *V. tuberculatum* as an alternative therapeutic source in the future. Therefore, it is necessary to collect and investigate the contributions of pure compounds for their therapeutic potential, including other human and animal diseases. As the existence of several species of parasitic plants, including *V. tuberculatum*, is threatened by various factors, there is a need for more attention to conserve these species for their ecological and therapeutic benefits in the future.
